# In-depth performance evaluation of PFP and ESG sequence-based function prediction methods in CAFA 2011 experiment

**DOI:** 10.1186/1471-2105-14-S3-S2

**Published:** 2013-02-28

**Authors:** Meghana Chitale, Ishita K Khan, Daisuke Kihara

**Affiliations:** 1 Department of Computer Science, Purdue University, 305 N. University Street, West Lafayette, Indiana, 47907, USA; 2 Department of Biological Sciences, Purdue University, 915 W. State Street, West Lafayette, Indiana, 47907, USA

## Abstract

**Background:**

Many Automatic Function Prediction (AFP) methods were developed to cope with an increasing growth of the number of gene sequences that are available from high throughput sequencing experiments. To support the development of AFP methods, it is essential to have community wide experiments for evaluating performance of existing AFP methods. Critical Assessment of Function Annotation (CAFA) is one such community experiment. The meeting of CAFA was held as a Special Interest Group (SIG) meeting at the Intelligent Systems in Molecular Biology (ISMB) conference in 2011. Here, we perform a detailed analysis of two sequence-based function prediction methods, PFP and ESG, which were developed in our lab, using the predictions submitted to CAFA.

**Results:**

We evaluate PFP and ESG using four different measures in comparison with BLAST, Prior, and GOtcha. In addition to the predictions submitted to CAFA, we further investigate performance of a different scoring function to rank order predictions by PFP as well as PFP/ESG predictions enriched with Priors that simply adds frequently occurring Gene Ontology terms as a part of predictions. Prediction accuracies of each method were also evaluated separately for different functional categories. Successful and unsuccessful predictions by PFP and ESG are also discussed in comparison with BLAST.

**Conclusion:**

The in-depth analysis discussed here will complement the overall assessment by the CAFA organizers. Since PFP and ESG are based on sequence database search results, our analyses are not only useful for PFP and ESG users but will also shed light on the relationship of the sequence similarity space and functions that can be inferred from the sequences.

## Background

New technologies have resulted in the abundance of sequence data that needs to be assigned with functional annotations. For example, the number of completely sequenced genomes in KEGG [1] database has more than doubled from 2007 (634 genomes) to 2012 (1646 genomes). This rapid growth in the sequenced data coupled with the lack of human resources to manually curate these genomes have resulted into the need to develop computational function annotation techniques [2-6]. The Automatic Function Prediction (AFP) community is attempting to bridge this gap by developing techniques that predict function annotations for proteins. In addition to conventional homology search methods, e.g. BLAST [7], FASTA [8], SSEARCH [9], and motif/domain searches, e.g. PRINTS [10], ProDom [11], Pfam [12], InterPro [13], and BLOCKS [14], several advanced methods were developed that extract function information thoroughly from sequence database search results. These methods include PFP [15,16], ESG [17], GOtcha [18], GOPET [19], OntoBlast [20], GOFigure [21], and ConFunc [22]. On the other hand, SIFTER [23], FlowerPower [24], and Orthostrapper [25] employ phylogenetic trees to transfer functions to target genes in the evolutionary context. There are other function prediction methods considering co-expression patterns of genes [26-30], 3D structures of proteins [31-39] as well as interacting proteins in large-scale protein-protein interaction networks [40-45].

For the advancement of such computational techniques it is very important that there are community wide efforts for objective evaluation of prediction accuracy. Community-wide prediction assessments have become popular in several computational prediction areas. Such experiments include CASP (Critical Assessment of techniques for Structure Prediction) [46] CAPRI (Critical Assessment of PRediction of Interactions) [47], and CAGI (Critical Assessment of Genome Interpretation) (http://cagi2010.org/). For the field of AFP, some experiments have been held in the past, which include MouseFunc 2006 (http://hugheslab.med.utoronto.ca/supplementary-data/mouseFunc_I/), ISMB (Intelligent Systems in Molecular Biology) AFP SIG (Special Interest Group) 2005 [48], the 2006 AFP meeting [49], and also the function prediction category in CASP6 [50] and CASP7 [51]. As a part of recently concluded ISMB conference 2011, CAFA (Critical Assessment of Function Prediction) experiment was conducted to gauge the Gene Ontology (GO) [52] prediction accuracy of various AFP methods (http://biofunctionprediction.org/).

In the CAFA experiment in 2011, in total of 48,298 target protein sequences were released for prediction, which consist of seven eukaryotic genomes, eleven prokaryotic genomes, and a supplementary set of additional sequences. The participating predictor groups were expected to submit GO annotations for these targets in Biological Process (BP) and Molecular Function (MF) domains. Out of these set, the organizers selected 436 targets in BP domain and 366 targets in MF domain that newly obtained experimental annotation in the SWISS-PROT database from January to May 2011, which is after the closing of the submission. Submitted predictions were evaluated using different prediction accuracy measures described in Methods.

We have submitted predictions using two methods developed in our group, the Protein Function Prediction (PFP) method [15,16] or the Extended Similarity Group (ESG) method [17]. PFP and ESG use PSI-BLAST sequence database search results, from which function information is extracted extensively, even from weakly similar sequences. In this article, we analyze the prediction performance of these two methods in comparison with BLAST, the Prior method, and GOtcha [18], whose predictions are provided by the CAFA organizers. Prediction performance evaluation employed four metrics used by the organizers; the threshold method, the top N method, the weighted threshold method, and the semantic similarity method (see Methods). Besides evaluating original predictions by PFP and ESG submitted to CAFA, we further investigated the following to have a better understanding of their performance: 1) For PFP predictions, we reranked predicted GO terms using a different score from the originally used score and compared the performances; 2) We combined PFP and ESG predictions with those from the Prior method that simply ranks GO term by the background frequency in a database; 3) We evaluated prediction accuracies of each method separately for different functional categories; and 4) We examined successful and unsuccessful predictions by PFP and ESG in comparison with BLAST. The in-depth analysis discussed here will complement the overall assessment by the CAFA organizers that will be published elsewhere. Since PFP and ESG are based on sequence database search results, our analyses are not only useful for PFP and ESG users but will also shed light on the relationship of the sequence similarity space and functions that can be inferred from the sequences.

## Methods

### Function prediction methods

In this section we briefly describe the AFP methods that are compared in this study. First we explain the PFP and ESG methods. Then BLAST, the Prior method, and GOtcha, whose prediction results were provided by CAFA organizers, are also described. Predictions in the MF and the BP domain were evaluated by comparing them with annotations with experimental evidences (i.e. non Inferred Electronic Annotations; non-IEA) in the UniProt database. For each target, predictions were restricted to 1000 highest score predictions with the score ranging between 0 and 1.

#### Protein function prediction (PFP) algorithm

The PFP algorithm [15,16] uses PSI-BLAST to obtain sequence hits for a target sequence and computes the score to GO term *f_a _*as follows:

(1) $$s\left(fa\right)=\overset{N}{\underset{i=1}{\sum }}\overset{Nfunc\left(i\right)}{\underset{j=1}{\sum }}\left(\left(-\text{log}\left(Evalue\left(i\right)\right)+b\right)P\left(fa|fj\right)\right),$$

where *N *is the number of sequence hits considered in the PSI-BLAST hits, *Nfunc(i) *is the number of GO annotations for the sequence hit *i, E-value(i) *is the PSI-BLAST E-value for the sequence hit *i, f_j _*is the *j*-th annotation of the sequence hit *i*, and constant *b *takes value *2 (= log_10_100) *to keep the score positive when retrieved sequences up to E-value of 100 are used. The conditional probabilities *P(f_a_|f_j_) *are to consider co-occurrence of GO terms in single sequence annotation, which are computed as the ratio of number of proteins co-annotated with GO terms *f_a _*and *f_j _*as compared with ones annotated only with the term *f_j_*. To take into account the hierarchical structure of the GO, PFP transfers the raw score to the parental terms by computing the proportion of proteins annotated with *f_a _*relative to all proteins that belong to the parental GO term in the database. The score of a GO term computed as the sum of the directly computed score by Eqn. 1 and the ones from the parental propagation is called the raw score.

In addition to the raw score, we also compute the p-value and the confidence score for a GO term. The purpose of computing the p-value of a GO term is to consider the background distribution of the raw scores of the GO term. It is computed using a term specific raw score distribution obtained from predictions made for protein sequences in a benchmarking dataset of eleven genomes. Then, the confidence score is further computed from the p-value by considering the percentage of correct predictions at each p-value within 0, 2, and 4 edge distance of the target term on the GO structure. In CAFA, predicted GO terms by PFP were sorted by the confidence score.

#### Extended Similarity Group (ESG) algorithm

ESG [17] recursively performs PSI-BLAST searches from sequence hits obtained in the initial search from the target sequence, thereby performing multi-level exploration of the sequence similarity space around the target protein. Each sequence hit in a search is assigned a weight that is computed as the proportion of the -log (E-value) of the sequence relative to the sum of -log (E-value) from all the sequence hits considered in the search of the same level; and this weight is assigned for GO terms annotating the sequence hit. The weights for GO terms found in the second level search are computed in the same fashion. Ultimately the score for a GO term is computed as the total weight from the two levels of the searches. The score for each GO term ranges from 0 to 1.0.

#### The prior method

In the prior method, each GO term is assigned the frequency of its occurrence in SWISS-PROT (January 2011 version) including a pseudo count of 1. For a given target sequence, top 1000 GO terms with highest frequencies were selected as predictions. Thus, all target sequences have the same set of predictions by this method. The prior predictions for each target were provided by the organizers.

We have also combined the prior predictions with predictions by PFP and ESG. These predictions are called the enriched PFP/ESG or PFP/ESG + Prior. In PFP + Prior, we added GO terms to PFP predictions that were not predicted by PFP (the expected accuracy was used for the PFP score). The score (i.e. frequency) for GO terms imported from the prior method was rescaled by considering maximum and minimum scores of PFP predictions for that target. GO terms originally predicted by PFP and ones imported from the prior method were sorted by the score. Similar to the PFP + Prior, ESG + Prior also combined the original ESG predictions and GO terms from the prior method that were not predicted by ESG. Since both the ESG score and the frequency in the prior method range from 0 to 1, GO terms from the two methods were sorted by the score without rescaling.

#### BLAST

BLAST search [7] with default parameters was performed for each target sequence. The score for a particular annotation term was the maximum sequence identity with the hit annotated with that term. Predictions by BLAST were provided by the organizers.

#### GOtcha

GOtcha [18] incorporates the hierarchical structure of GO vocabulary with the idea of homology based annotation transfer to achieve improved coverage. It uses BLAST [7] to search similar sequence hits and assigns a score, -log(E-value), to each GO annotation of the sequence hits and its less specific ancestors in the GO hierarchy. The scores assigned to each GO node from all the sequence hits are summed and then normalized using the score of the root of either MF or BP ontology. The normalized score thus obtained is referred as I-score, which was used for selecting target annotations. Predictions by GOtcha were provided by the organizers.

### Assessment methods for prediction accuracy

In CAFA, predictions were evaluated using four different methods. The threshold and the top N methods count exact match of predicted and the actual annotations, punishing any predictions that are more or less specific than the actual annotations. On the other hand, the weighted threshold and the semantic similarity take into account the information content of terms being matched on the GO hierarchy. Please refer to the organizers' paper in the same journal issue for more details. We have used Gene Ontology version October 2011 for obtaining ancestors for each GO term.

#### Threshold method

For each prediction method we use thresholds ranging from 0.01 to 1.0 to calculate the average precision, recall, and specificity for all targets. For each target if a particular prediction has a score above the threshold, the predicted GO term is propagated to the root of the ontology. The performances are analyzed in terms of precision-recall curve and the receiver operator characteristic (ROC). For the threshold method, when using PFP raw scores that are not scaled between 0 and 1, we selected 1 to 1000 GO term predictions by the increments of 5 and compute average precision, recall and specificity for all targets.

#### Top N

The top N highest scoring predictions for a prediction method are taken into consideration with N varying from 1 to 20. For all the predictions within top N, parental annotations until the root of the ontology are included. All predicted annotations with a tie score at a particular ranking are considered for the cutoff.

#### Weighted threshold

As shown in Equation 2, frequency of a GO term *c *in the database is computed as the number of gene products annotated by term *c *and its children *h *in the GO hierarchy.

(2) $$freq\left(c\right)=annot\left(c\right)+\underset{h\in child\left(c\right)}{\sum }freq\left(h\right),$$

where *annot(c) *is the number of gene products annotated by non IEA evidence codes in September 2011 version of SWISS-PROT database. Probability of a particular term *c*, *p(c) = freq(c)/freq(root)*, is computed as the ratio of the frequency of *c *against the frequency of the root term of the MF or BP ontology. Information content of term *c *is given by *IC(c) = -log_10_(p(c))*. Using this information content, weighted precision is calculated as the sum of information content of the terms in the true positive set divided by the sum of information content of the terms in the true and false positive sets. Similarly, weighted recall is computed as the sum of information content of the terms in the true positive set divided by the sum of information content of the terms in the true positive and false negative sets. As with the previous methods, if a particular prediction is above the given threshold, then its ancestors till the root of the ontology are included in the prediction set.

#### Semantic similarity

Semantic similarity for a pair of GO terms is given by the maximum information content of a shared ancestor of both terms and it is averaged between all pairs of true and predicted terms to obtain the semantic similarity for a target. We calculate the semantic precision for a target protein as the average of the difference between the IC of a predicted term and the maximum of the IC of common parental terms between the predicted term and any correct term. Similarly, semantic recall is calculated for a target as the average of the difference between the IC of a true term and the maximum of the IC of common parental terms between the true term and any predicted term. Here the information content values are based on the Prior probabilities for each term provided by the CAFA organizers. The average semantic similarity, semantic precision and semantic recall are computed across all targets at each threshold varying from 0.01 to 1.0.

## Results

### PFP with raw scores

In the CAFA experiment we submitted PFP predictions sorted by the confidence score. In this section, we ranked predicted GO terms by PFP according to the raw score and examined how its performance compared with the confidence score and the other methods. From ranked list of PFP predictions by their raw score, precision, recall, and specificity were calculated at each of the top N predictions taken with an interval of 5.

**Figure 1:**
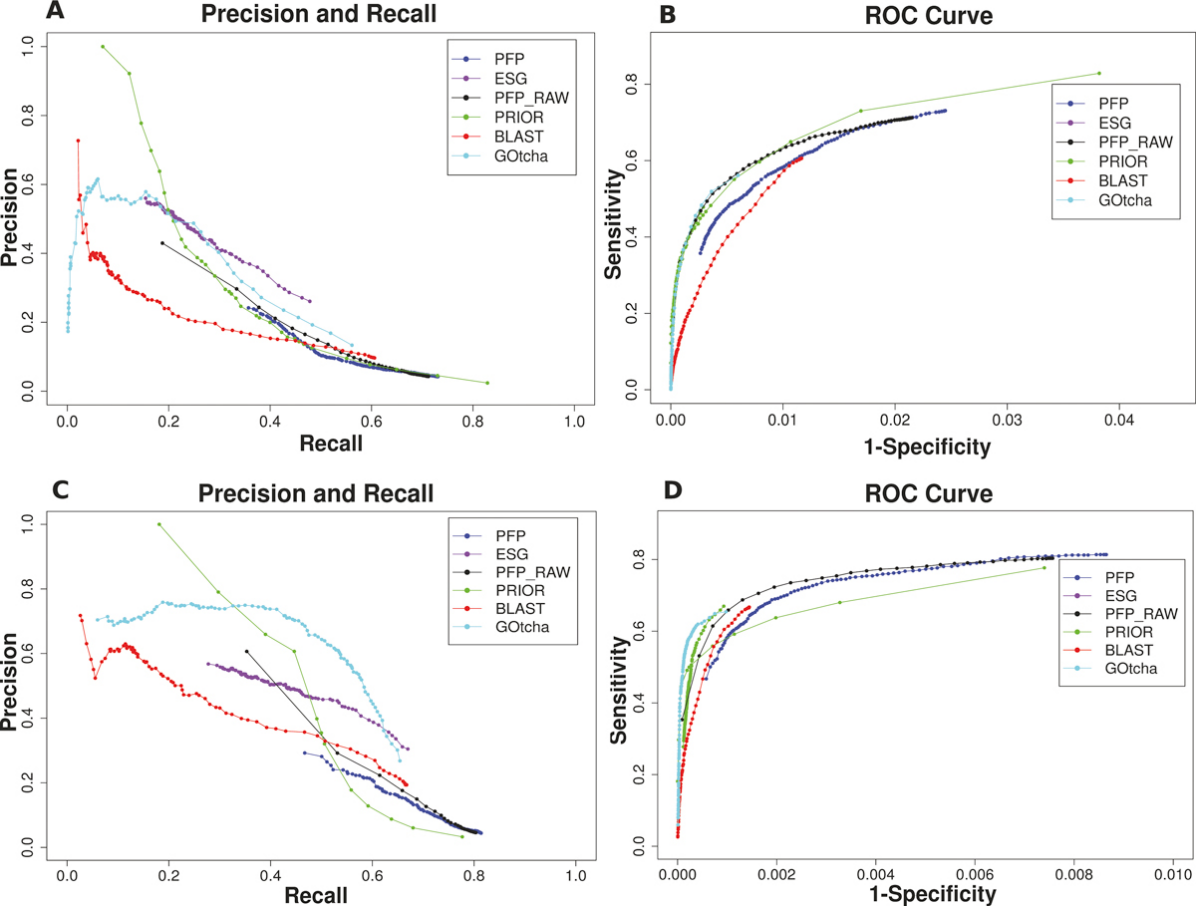
**Performance of PFP (confidence score), PFP prediction sorted by the raw score (PFP_RAW), ESG, PRIOR, BLAST, and GOtcha**. **A**, Precision - Recall plot for the BP domain. **B**, ROC for the BP domain. **C**, Precision - Recall plot for the MF domain. **D**, ROC for the MF domain.

Figure 1 shows the precision-recall curve and the ROC of PFP with raw score compared with the other methods. For the BP domain, we observe that PFP with raw score (PFP_RAW in the plots) has slightly higher precision for a given recall value than PFP predictions ranked by the confidence score (PFP). PFP with raw score has clearly better performance than PFP with confidence score in the ROC curve (Figure 1B), particularly at lower false positive range (x-axis). The similar behavior of PFP raw score is observed for predictions in the MF domain (Figure 1C &1D). These results indicate that the confidence score of PFP, which is computed in two steps from the raw score via the p-score distribution (see Methods), was not very successful in ranking predicted GO terms especially at top ranks (lower false positive regions). Thus, derivation of the confidence score need s to be reexamined and probably revised.

### PFP and ESG with enriched priors

**Figure 2:**
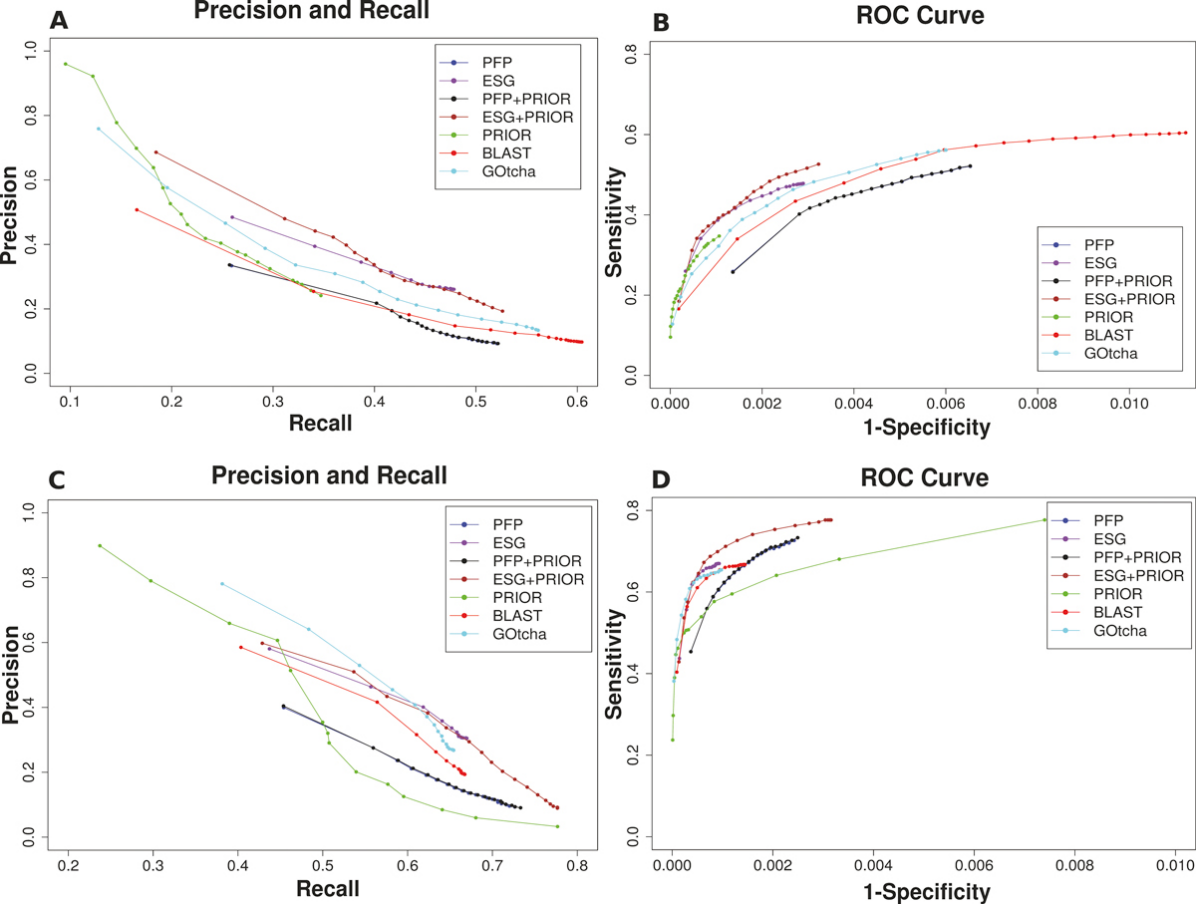
**Performance of PFP and ESG with enriched priors (PFP/ESG+PRIOR), PFP, ESG, Prior, BLAST and GOtcha**. The top N method was used for this evaluation. **A**, Precision - Recall plot for the BP domain; **B**, ROC for the BP domain; **C**, Precision - Recall plot for the MF domain; **D**, ROC for the MF domain.

Next, we combined the PFP and ESG predictions with the prior predictions (PFP + Prior, ESG + Prior) to see if PFP/ESG predictions were missing obvious GO terms (Figure 2). We show the performance of the methods is evaluated with the top N method, where N ranges from 1 to 20.

ESG with enriched priors (ESG + Prior) shows the best performance among all the methods in BP domain when evaluate by the precision-recall plot (Figure 2A). The improvement by ESG + Prior over ESG is also observed in terms of ROC (Figure 2B). ESG + Prior also performed better than ESG in the MF domain (Figures 2C &2D). ESG tends to predict fewer GO terms than even BLAST since its algorithm essentially selects terms that are consistently identified by iterative searches. The results in Figure 2 indicate that obvious GO terms in Prior were not included in ESG predictions. Since some GO terms may be lost in the iterative process of the ESG algorithm, the scoring scheme needs to have a close inspection as a future work. On the other hand, adding Prior prediction to PFP did not show any improvement over PFP, which indicates that PFP's predictions already include correct terms from Prior.

### PFP and ESG with semantic similarity

**Figure 3:**
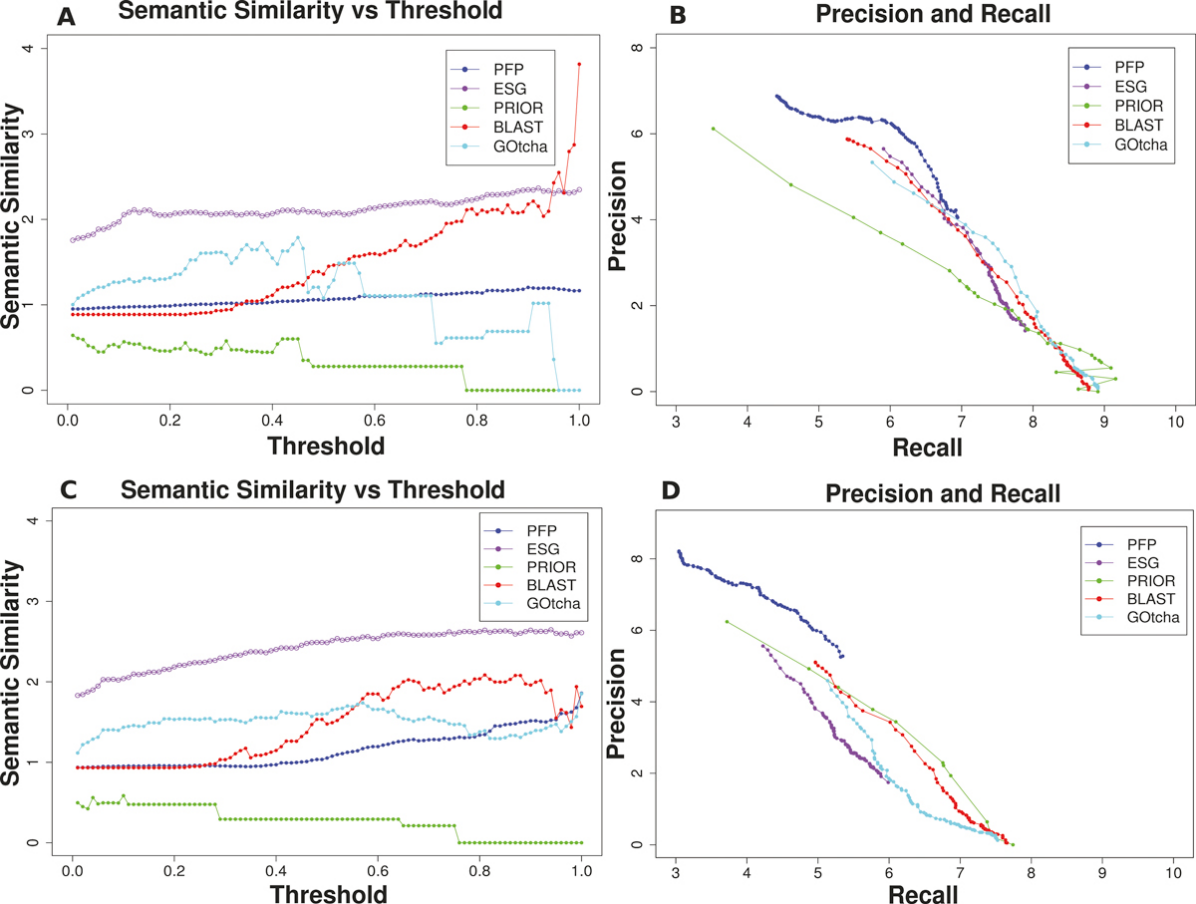
**Performance of PFP and ESG as compared with Prior, BLAST, and GOtcha using semantic similarity method**. **A**, Semantic similarity relative to the score threshold. Predictions in the BP domain are evaluated; **B**, semantic precision vs semantic recall for the BP domain; **C**, Semantic similarity relative to the score threshold in the MF domain; **D**, semantic precision vs semantic recall for the MF domain.

In Figure 3 the performance of the methods are evaluated in terms of the semantic similarity. The average of the semantic similarity between all pairs of true and predicted GO terms for each method is plotted relative to thresholds in Figure 3A and 3C for the BP and MF domain, respectively. It is shown that ESG's performance is significantly better than the other methods for both BP and MF targets. PFP performance is average among all the teams in this measure. On the other hand, PFP stands out in the semantic precision and recall plots (Figures 3B &3D). ESG comes second in the BP domain (Figure 3B) but shows worst performance among all in the prediction of MF terms (Figure 3D).

Prediction accuracy for different GO terms

**Figure 4:**
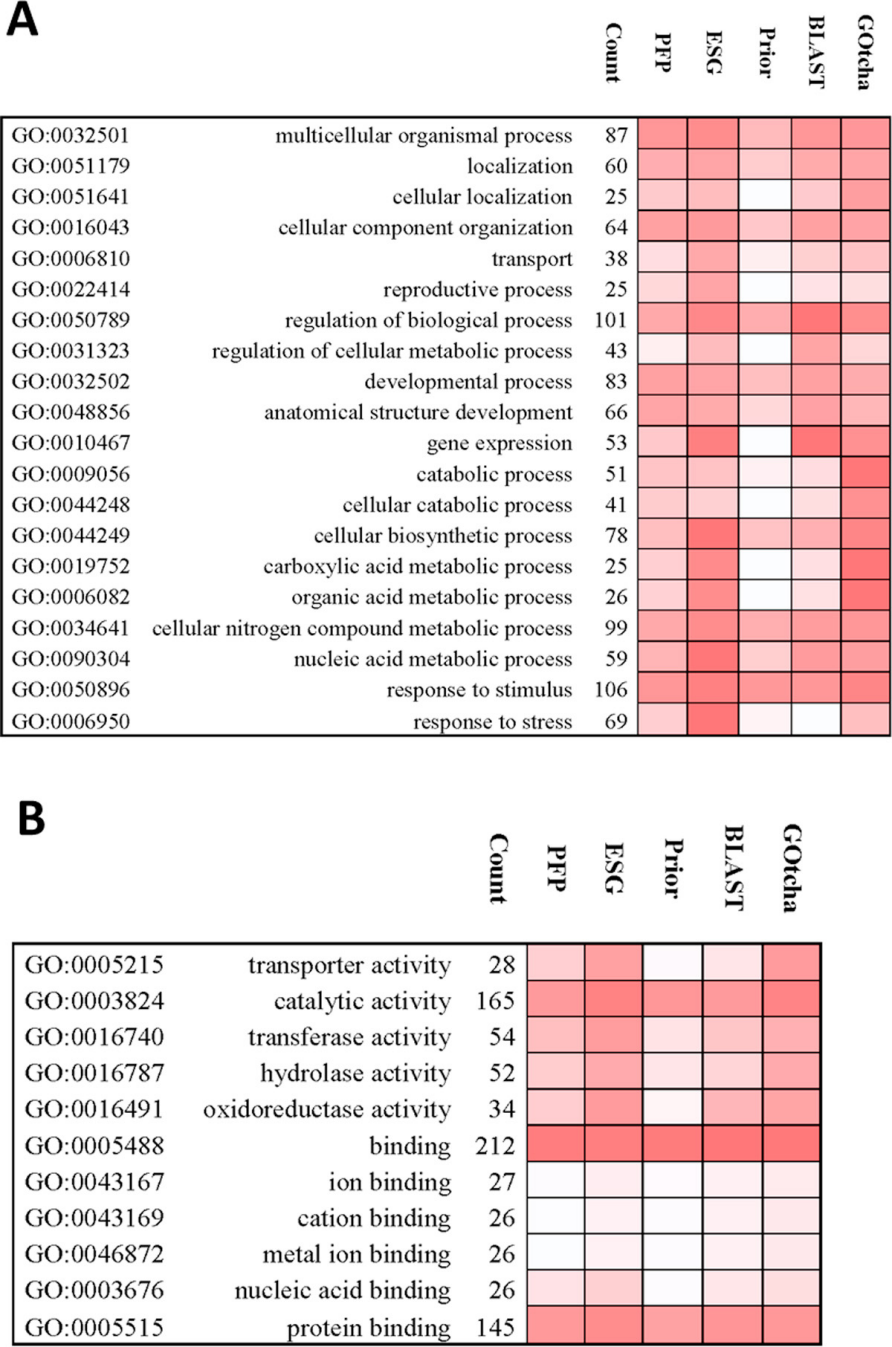
**Prediction accuracy evaluated for each functional category**. Each row represents a GO term category and each column represents a prediction method. Count refers to the number of target proteins that were annotated by the given a GO term in the category. The F1 measure was used for evaluation. The color ranges from white (minimum) to red (maximum score). **A**, the BP domain. Results of a sample of 20 terms are shown, which are taken out of the 77 BP terms annotating 25 or more targets. **B**, the MF domain. Results for 11 MF terms each annotating 25 or more targets are shown.

In Figure 4, we analyze the prediction accuracy for different GO terms. Only GO terms and their child terms that are used for annotating 25 or more targets are considered. This results in 77 BP terms and 11 MF terms for this analysis.

For each GO term under consideration, we identified target proteins that are annotated by the GO term and counted how many were correctly predicted to have the same annotation by each of the prediction methods. For example, there were 38 out of 436 BP targets that were annotated by the BP GO term *GO:0006810 transport*. The number of targets out of 38 that were predicted by a method to have the same annotation were considered as true positives (TP) and the targets that were not predicted were considered as false negatives (FN). The rest of the 398 targets that do not have actual annotation (*GO:0006810 transport*) but were predicted to have this annotation were considered as false positives (FP). To take into account of the fact that parental terms of a GO term were less specific descriptions of the same function, we have included all the ancestors of each predicted term shortlisted based on the cutoff score used. For each selected term at the cutoffs ranging from 0.01 to 1.00, precision, TP/(TP+FP), and recall, TP/(TP+FN), were computed. Further, F1 measure for the term is calculated as the maximum harmonic mean at each cutoff, which is given by

(3) $$F1=\frac{2^{*}\text{Pr}ecision^{*}\text{Re}call}{\text{Pr}ecision+\text{Re}call}$$

We compared PFP (using the confidence score) and ESG predictions submitted to CAFA, BLAST, Priors, GOtcha and enriched PFP and enriched ESG predictions.

Out of the 77 BP terms, ESG showed the highest F1 measure among the seven methods for 31 terms. PFP, Priors, BLAST, GOtcha, enriched PFP, and enriched ESG showed highest F1 measure for 3, 1, 22,20, 3, and 19 (ESG has 19 ties with enriched ESG and PFP has 3 ties with enriched PFP) terms, respectively. The average F1 measures across the 77 BP terms by PFP, ESG, Prior, BLAST, GOtcha, enriched PFP, and enriched ESG were 0.288, 0.367, 0.222, 0.329, 0.329, 0.291, and 0.342, respectively. In Figure 4A a sample of 20 BP terms out of 77 is shown. For the term *GO:0006950 response to stress *that is used to annotate 69 proteins, ESG showed the best F1 measure value of 0.36 followed by PFP (0.30) and GOtcha (0.31). On the other hand, for *GO:0016043 cellular component organization *that shares annotations with 64 BP targets, all five methods except Priors and enriched ESG showed comparable performance with F1 measure around 0.35. Overall, the enriched PFP method showed almost identical F1 scores to PFP whereas the enriched ESG showed slightly lower average F1 score as compared to ESG. Enriched ESG showed a higher recall than ESG but with a lower precision, which overall decreased the F1 score than ESG.

Figure 4B shows the results for the eleven MF terms. In six out of eleven terms annotating more than 25 targets, ESG showed the best F1 measure. PFP, Prior, BLAST, GOtcha, enriched PFP, and enriched ESG had the highest F1 score for 0, 0, 1, 5, 0, and 4 categories, respectively (ESG, enriched ESG, and GOtcha have tie for one case as well as ESG and enriched ESG tie for 4 cases). The average F1 scores across the eleven terms for PFP, ESG, Prior, BLAST, GOtcha, enriched PFP, and enriched ESG are 0.374, 0.484, 0.298, 0.386, 0.474, 0.375, and 0.474 respectively. Thus, overall ESG performed best for both BP and MF domains. In this analysis (Figure 4), generally it is observed for both BP and MF domains that all sequence database search-based methods showed higher average F1 scores than Prior for most of the terms. Also for many terms, PFP, ESG, and GOtcha, which are built on (PSI-)BLAST performed better than BLAST.

### Examples of successful and failed of PFP and ESG predictions

Finally, we discuss the prediction examples (Table 1) where PFP, ESG, and BLAST succeeded at different levels that provide insights into the advantages and shortcomings of our methods. Each case in Table 1 shows correct target annotations propagated till the root of the ontology which were predicted by PFP, ESG, and BLAST. Since the number of actual and predicted GO terms for a target can be very large (over 100) when predictions of low scores are included, Table 1 includes only terms that are relevant to discussion.

**Table 1:** CAFA target prediction examples for PFP, ESG, and BLAST

CAFA Target		GO Term	Definition	Score
T06450, *E. coli *A34 Protein trbA *F1 measure* PFP:0.9 ESG:0.2 BLAST:0	**Selected CAFA Target** **Annotations**	GO:0008152 GO:0044238 GO:0044237 GO:0043170 GO:0044260 GO:0019538 GO:0044267 GO:0006457 GO:0042026	metabolic process primary metabolic process cellular metabolic process macromolecule metabolic process cellular macromolecule metabolic process protein metabolic process cellular protein metabolic process protein folding protein refolding	
	
	**Selected PFP Annotations**	GO:0044260 GO:0015804 GO:0046483 GO:0009110 GO:0019538 GO:0009110 GO:0019538	cellular protein metabolic process carboxylic acid metabolic process cellular macromolecule metabolic process neutral amino acid transport heterocycle metabolic process vitamin biosynthetic process protein metabolic process	0.99 0.81 0.81 0.8 0.8 0.77 0.77
	
	**Selected ESG Annotations**	GO:0000746 GO:0006810 GO:0008643 GO:0007165 GO:0008152 GO:0008652	conjugation transport carbohydrate transport signal transduction metabolic process amino acid biosynthetic process	0.61 0.2 0.12 0.09 0.05 0.05
	
	**Selected BLAST** **Annotations**			

T06299, *E. coli *rutE *F1 measure* PFP:0.18 ESG:0.13 BLAST:0	**Selected CAFA Target** **Annotations**	GO:0019740 GO:0008152 GO:0034641 GO:0006139 GO:0006206 GO:0019860 GO:0006208 GO:0006212	nitrogen utilization metabolic process cellular nitrogen compound metabolic process nucleobase, nucleoside, nucleotide and nucleic acid metabolic process pyrimidine base metabolic process uracil metabolic process pyrimidine base catabolic process uracil catabolic process	
	
	**Selected PFP Annotations**	GO:0006139 GO:0046131	nucleobase, nucleoside, nucleotide and nucleic acid metabolism pyrimidine ribonucleoside metabolism	1 1
	
	**Selected ESG Annotations**	GO:0055114	oxidation-reduction process	1
	
	**Selected BLAST** **Annotations**			

T05345, *E. coli *Sensor protein CpxA *F1 measure* PFP:0.32 ESG:0.68 BLAST:0.43	**Selected CAFA Target** **Annotations**	GO:0008152 GO:0044237 GO:0006796 GO:0016310 GO:0044260 GO:0006464 GO:0043687 GO:0006468 GO:0046777	metabolic process cellular metabolic process phosphate metabolic process phosphorylation cellular macromolecule metabolic process protein modification process post-translational protein modification protein amino acid phosphorylation protein amino acid auto phosphorylation	
	
	**Selected PFP Annotations**	GO:0007165 GO:0016310 GO:0006139 GO:0019222 GO:0043283	signal transduction phosphorylation nucleobase, nucleoside, nucleotide and nucleic acid metabolism regulation of metabolism biopolymer metabolism	1 1 1 1 1
		
		GO:0006464 GO:0006468 GO:0015698	protein modification protein amino acid phosphorylation inorganic anion transport	0.99 0.99 0.99
	
	**Selected ESG Annotations**	GO:0000160 GO:0007165 GO:0018106 GO:0016310 GO:0009405 GO:0006950	two-component signal transduction system (phosphorelay) signal transduction peptidyl-histidine phosphorylation phosphorylation pathogenesis response to stress	1 1 1 0.93 0.39 0.33
	
	**Selected BLAST** **Annotations**	GO:0000160 GO:0006950 GO:0009987 GO:0009628 GO:0006979 GO:0044260 GO:0043687 GO:0006468 GO:0046777	two-component signal transduction system (phosphorelay) response to stress cellular process response to abiotic stimulus response to oxidative stress cellular macromolecule metabolic process post-translational protein modification protein amino acid phosphorylation protein amino acid auto-phosphorylation	0.39 0.39 0.39 0.39 0.39 0.28 0.28 0.28 0.25

T18799, *Homo sapiens *Ribonuclease H2 subunit B *F1 measure* PFP:0.5 ESG:0.47 BLAST:0.79	**Selected CAFA Target** **Annotations**	GO:0006139 GO:0006401 GO:0006401 GO:0044248 GO:0034641 GO:0044260 GO:0090304	nucleobase, nucleoside, nucleotide and nucleic acid metabolic process RNA catabolic process RNA catabolic process cellular catabolic process cellular nitrogen compound metabolic process cellular macromolecule metabolic process nucleic acid metabolic process	
	
	**Selected PFP Annotations**	GO:0050789 GO:0044267 GO:0046451 GO:0006351 GO:0044260 GO:0006721 GO:0044238 GO:0016070	regulation of biological process cellular protein metabolism diaminopimelate metabolism transcription, DNA-dependent cellular macromolecule metabolism terpenoid metabolism primary metabolism RNA metabolism	1 0.9 0.86 0.83 0.83 0.82 0.81 0.41
	
	**Selected ESG Annotations**	GO:0006412 GO:0006418 GO:0006429	translation tRNA aminoacylation for protein translation leucyl-tRNA aminoacylation	0.02 0.01 0.01
	
	**Selected BLAST** **Annotations**	GO:0006139 GO:0006259 GO:0006260 GO:0006261 GO:0006271 GO:0006401 GO:0006807	nucleobase, nucleoside, nucleotide and nucleic acid metabolic process DNA metabolic process DNA replication DNA-dependent DNA replication DNA strand elongation involved in DNA replication RNA catabolic process nitrogen compound metabolic process	0.4 0.4 0.4 0.4 0.4 0.4 0.4

This table shows partial list of GO annotations and their ancestors for four targets that have been predicted using PFP, ESG, and BLAST. The first column lists the target IDs along with the maximum F1 score among predictions at all cutoffs using the predicted terms and their ancestors from each method. For each method we list the predicted GO annotations and partial list of their ancestors along with their scores.

The first example is T06450, *Escherichia coli *protein trbA, which is annotated with *GO:0042026 protein refolding *as per the CAFA target annotations. BLAST search finds only one sequence hit O26024 that does not have any non-IEA annotation in the database resulting in no predictions. As for ESG, some of the correct low resolution annotations are extracted from a sequence hit Q9UZ03 retrieved in the first iteration of PSI-BLAST search with very large E-value (above 1) and its second level hits, including Q8A608, Q64PS6, Q5L9I8. These predicted annotations are parental terms of actual annotations. For example, a predicted term, *GO:0008152 metabolic process*, is a parental term of *GO:0042026 protein refolding*, and *GO:0008652 amino acid biosynthetic process *shares a common ancestor *GO:0044237 cellular metabolic process *with the target annotation *GO:0042026 protein refolding*. PFP was able to predict some low resolution parental terms of the correct annotation such as *GO:0046483 cellular macromolecule metabolic process *and *GO:0044260 cellular protein metabolic process*, with significantly high confidence scores of 0.81 and 0.99. Both these terms are not part of annotations of any of the PSI-BLAST hit but received partial scores by considering co-occurrence of GO terms (i.e. *P(f_a_|f_j_) *in Eq. 1).

The second example, T06299, rutE from *E. coli*, is annotated by two leaf terms *GO:0019740 nitrogen utilization *and *GO:0019860 uracil metabolic process*. For this target BLAST again does not predict anything as there are no search hits with non IEA annotations. Using IEA annotation of highly similar PSI-BLAST hits, ESG predicted *GO:0055114 oxidation-reduction *process, which shares a shallow common ancestor *GO:0008152 metabolic process *with a target term *GO:0006212 uracil catabolic process*. Similar to the previous example, PFP again predicted low resolution annotations *GO:0006139 nucleobase, nucleoside, nucleotide and nucleic acid metabolism *and *GO:0046131 pyrimidine ribonucleoside metabolism *thereby showing higher sensitivity when no close homologs are available for annotation transfer.

The third target T05345 is sensor protein CpxA from *E. coli *with leaf annotation *GO:0046777 protein amino acid autophosphorylation*. ESG predicted *GO:0018106 peptidyl-histidine phosphorylation*, which shares an immediate parent *GO:0006468 protein amino acid phosphorylation *with the target term *GO:0046777 protein amino acid autophosphorylation*. Also another term *GO:0016310 phosphorylation*, which is an ancestor of the target annotation is predicted by ESG with a high score of 0.93. PFP correctly predicts the ancestors of the target term, *GO:0016310 phosphorylation, GO:0006464 protein modification and GO:0006468 protein amino acid phosphorylation *with very high scores. BLAST predicts the target term and its ancestors with lower scores along with a number of unrelated predictions with high scores. Overall all the methods are able to predict the target term or its close ancestors, but the total number of terms predicted by BLAST (193 terms) and PFP (134 terms) are significantly higher than ESG (7 terms), resulting into more precise predictions by ESG.

The last example, T18799, *Homo sapiens *Ribonuclease H2 subunit B, is annotated by a leaf term *GO:0006401 RNA catabolic process *which has been accurately predicted by BLAST. BLAST obtains this correct annotation from sequence hits such as Q5TBB1, Q5XI96, Q3ZBI3, Q80ZV0, Q28GD9, and Q5HZP1. These sequences were also found by ESG, however, due to use of an older database that do not have updated annotations for these sequences, no correct annotation was retrieved. There are some shared ancestors, e.g. *GO:0016070 RNA metabolic process, GO:0090304 nucleic acid metabolic process, GO:0044260 cellular macromolecule metabolic process *between the low scoring ESG prediction *GO:0006429 leucyl-tRNA aminoacylation *and the target annotation *GO:0006401 RNA catabolic process*. PFP was able to correctly predict low resolution terms, *GO:0044260 cellular macromolecule metabolism *and *GO:0016070 RNA metabolism*.

To summarize, the first and the second examples illustrate a situation where PFP predicts low resolution parental terms of actual annotations while BLAST can only find 1 or 0 terms. There are PFP's successful prediction which were found indirectly by using the GO term co-occurrence. In the second example, IEA annotations lead to correct predictions for ESG and PFP. The third example is the case that ESG made predictions with higher precision with smaller number of false positives than BLAST and ESG. The last example is that ESG missed to make correct prediction because the sequence database which was searched was not up-to-date.

## Discussion

In this work we have analyzed the prediction performance of PFP and ESG in the CAFA 2011 experiment. In addition to the original submission of PFP and ESG, we have investigated the performance of a different scoring function (the raw score) for PFP, to examine if quick improvement is possible by considering prior knowledge of frequency of GO terms in the database (i.e. PFP + Prior, ESG + Prior). Moreover, we evaluated prediction accuracy at each functional categories and provided illustrative examples to understand successful and failed predictions by PFP and ESG.

Several points can be concluded from this study: Firstly, correct function information can be extracted from BLAST results more extensively than simply taking GO terms from top hits as demonstrated in some of the results that show superior performance by PFP, ESG, and GOtcha over BLAST. However, there are situations where PFP and ESG's performance compared unfavorably to BLAST and Prior depending on how performance is evaluated. Also we observed that ESG predictions are improved by simply adding Prior. Thus, we believe there is still room for improvement by devising techniques for consistently extracting accurate information thoroughly from (PSI-)BLAST results. It is observed that IEA terms provided correct information in several cases, which led to better performance by PFP/ESG over BLAST that only considered non-IEA hits. Keeping IEA information in the database would enlarge areas in the sequence similarity space with functional information, and thus increase the chance to retrieve correct function information, although one should always keep in mind that IEA may be incorrect, and moreover, careless applications of computational function prediction methods would increase incorrect IEA and propagate erroneous annotation through databases [53,54].

## Conclusion

We have analyzed function predictions by PFP and ESG that were submitted to CAFA 2011. Overall ESG and PFP showed better performance than BLAST and Prior, but there are also opposite situations. Some of the lessons learned would be generally useful for developers and users of computational function prediction methods.

## Competing Interests

The authors declare that they have no competing interests.

## Financial Competing Interests

The authors declare that they have no financial competing interests.

## Authors' Contributions

MC submitted predictions to CAFA. In addition, MC participated in design, implementation of evaluation programs for the study, performed the analysis, and drafted the paper. IK coded some of the evaluation programs, performed the analysis, and drafted the paper. DK conceived of the study, participated in its design, and finalized the manuscript. All authors read and approved the final manuscript.

## Declarations

This article has been published as part of *BMC Bioinformatics *Volume 14 Supplement 3, 2013: Proceedings of Automated Function Prediction SIG 2011 featuring the CAFA Challenge: Critical Assessment of Function Annotations. The full contents of the supplement are available online at URL. http://www.biomedcentral.com/bmcbioinformatics/supplements/14/S3.
